# Supplementary material for: Splice-Junction-Based Mapping of Alternative Isoforms in the Human Proteome
Source: Cell Rep. Author manuscript; Available in PMC 2020 Jan 15. (PMC6961840; doi:10.1016/j.celrep.2019.11.026)
Supplement: 2 [file NIHMS1546469-supplement-2.zip › DF1/PXD010154/Ovary/Ovary_1_PCBP2_SGLDASAQTTSHELTIPNDLIGCIIGR.pdf]

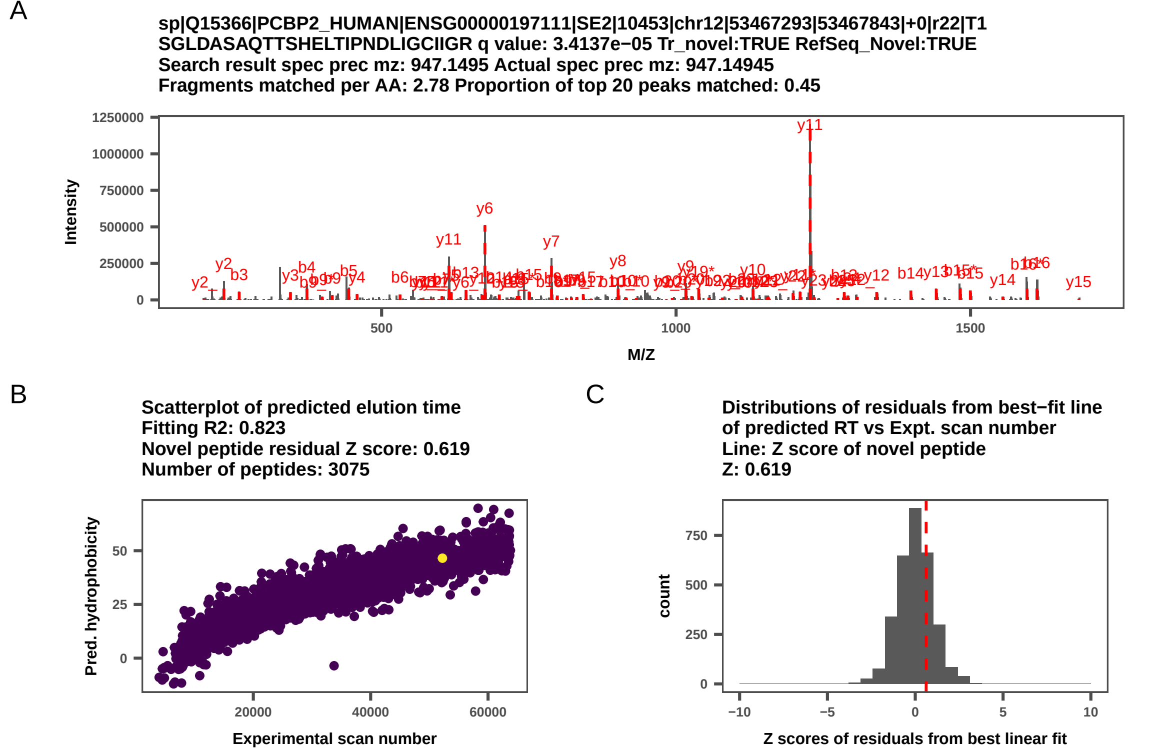
A
sp|Q15366|PCBP2_HUMAN|ENSG00000197111|SE2|10453|chr12|53467293|53467843|+0|r22|T1
SGLDASAQTTSHELTIPNDLIGCIIGR q value: 3.4137e−05 Tr_novel:TRUE RefSeq_Novel:TRUE
Search result spec prec mz: 947.1495 Actual spec prec mz: 947.14945
Fragments matched per AA: 2.78 Proportion of top 20 peaks matched: 0.45
Intensity
1250000
1000000
750000
500000
250000
0
y11
y6
y11
y7
y8
y2
b3
b4
b5
y4
y10
y12
b14
y14
y15
500
1000
1500
M/Z
B
Scatterplot of predicted elution time
Fitting R2: 0.823
Novel peptide residual Z score: 0.619
Number of peptides: 3075
Pred. hydrophobicity
50
25
0
20000
40000
60000
Experimental scan number
C
Distributions of residuals from best−fit line
of predicted RT vs Expt. scan number
Line: Z score of novel peptide
Z: 0.619
count
750
500
250
0
−10
−5
0
5
10
Z scores of residuals from best linear fit
